# An investigation of simulated and real touch on feelings of loneliness

**DOI:** 10.1038/s41598-023-37467-5

**Published:** 2023-06-30

**Authors:** Nicholas L. T. Gray, S. Craig Roberts

**Affiliations:** Department of Psychology, University of Stirling, Stirling, UK

**Keywords:** Psychology, Anthropology

## Abstract

As a social species, humans deprived of contact find loneliness a potentially distressing condition. Recent research emphasises the influence of touch on alleviating loneliness. This research found that touch reduces feelings of neglect, a subscale of loneliness. Affectionate touch, which demonstrates care or affection, has been previously linked to well-being in couples. Here, we investigated whether the effect of simulated touch during a video conversation might be sufficient to influence feelings of loneliness. Sixty participants answered a survey about their home life and relationships, including items that assessed the frequency of touch and feelings of loneliness. Following this, they participated in an online video call with three conditions: audio only, audio and video, or audio, video with simulated touch (a virtual ‘high-five’). Finally, immediately after the call, they repeated the loneliness questionnaire. We found that loneliness scores were reduced following the call, but there was no difference among conditions and no effect of a virtual touch. However, we did find a significant association between the frequency of touch in a relationship and the expression of loneliness, with individuals in low-touch relationships having loneliness scores more comparable to single participants than to those in high-touch relationships. Additionally, extraversion played a major role in moderating the effect of touch in relationships. These results emphasise the importance of physical contact in lowering feelings of loneliness within relationships and the ability of calls to lower feelings of loneliness, regardless of whether they include video or simulated touch.

## Introduction

Loneliness is a condition experienced by many people during at least some stage of life. If left unchecked, it has severe mental and physical health consequences, increasing the likelihood of death by up to 26%^1^. The prevalence of loneliness worsened during the COVID-19 pandemic, with over 1 million people feeling “often” or “always” lonely in the first year of the pandemic in Britain^2^.

Finding ways to ameliorate and prevent feelings of loneliness is, therefore, a pressing issue. A mistakenly simple intervention would be to reduce social isolation, as one theory of loneliness states that loneliness is an evolutionary trait “motivating reconnection with other people, increasing chances of survival”^3^. However, this is not necessarily an effective intervention for those struggling with loneliness^4^. Meanwhile, focusing on emotion management, social skills, anxiety, and negative cognitive biases has shown potential for intervention strategies^5^. Most importantly, loneliness intervention should be tailored to the individual’s needs^5^.

Despite requiring an individualistic approach, the World Health Organization (WHO) recommended keeping social connections active through digital means, such as phone and video calls to alleviate or prevent loneliness during the COVID-19 pandemic^6^. Supporting the WHO’s advice^6^, increased phone use was associated with decreased feelings of loneliness^7^. Furthermore, digital technology has also been found to play a critical role in managing overall mental well-being^8^.

However, when considering that part of loneliness is a deficit of specific relational provisions, such as touch^9^, the demand for a more holistic approach becomes apparent. Indeed, touch itself has also previously been linked to better physical and mental wellbeing^10,11^. This is perspicuous when considering that touch-based bonding mechanisms are deeply rooted in human evolution^11^. If viewing loneliness as an embodied and contextualised sensory experience, the relationship with touch is clear^12^. Furthermore, the demand for more research investigating the interaction of touch and loneliness has grown^12^.

Most research involving touch and well-being used affectionate touch, defined as physical contact demonstrating love, care, fondness, appreciation, or something assumed to indicate affection^10^. Therefore, all cases of touch discussed in this introduction should be seen as affectionate touch unless otherwise stated.

The influence of touch on perceived loneliness was demonstrated in an experimental context by Heatley Tejada et al.^13^. Participants assigned to a treatment condition had baby oil massaged onto the back of their hand by an experimenter, ostensibly to prepare for assessment of their autonomic balance in a laboratory task. In the control group, participants were asked to apply the oil themselves. Subsequent completion of a brief loneliness scale revealed lower scores of neglect in those in the treatment group, which were attributed to the touch by the experimenter^13^. Supportively, daily embraces were found to reduce the negative effects of loneliness on mood^15^. Another study found that the mere sight of a leg being touched activated the same region of participants’ brains as when the person was physically touched, suggesting the sight of touch may provide similar results to actual touch^14^.

Additionally, the observation of sensory experiences was found to have the potential to activate mirror neurons and sensorimotor-related brain regions^16^. Thanks to the human ability to release endorphins and bond through non-touch behaviours, such as laughter and a range of activities, activating these brain regions may help reduce loneliness without direct touch^17^. Beyond bonding and touch, children who received praise in the form of a high five (touch) or a thumbs up (gestural) perceived both the same way and felt significantly better about themselves^18^. This demonstrates the similarities and power of touch and visual cues. In sum, touch has been found to lower feelings of loneliness. At the same time, additional research suggests these effects can be transferable to a simulated or digital touch to the effects of touch being transferable to video.

In addition to transferring the effects of touch on loneliness to call, relationships need to be considered. Some evidence suggests that touch with a stranger has reduced threat regulation compared to touch with a stranger^19^, with recent evidence suggesting it might have no effect^20^. However, when discussing loneliness, a welcomed touch from a stranger has been shown to lower feelings of loneliness^12,13^. Support from weak ties can also be effective, but work is needed to establish the situations in which this is true^21^. Since loneliness can be seen as a deficit of necessary relational provisions^9^, support from weak ties may help to fulfil this deficit.

Relationship status may also influence loneliness, of course, but it may also moderate the effect of touch. Heatley Tejada et al.^13^ also reported that, while participants in a relationship had lower scores of neglect regardless of whether they were in the experimental or control group (i.e., receiving a touch from the experimenter or not), single participants subjected to touch reported significantly lower neglect scores than those who had no touch^13^. Based on this, Heatley Tejada et al. concluded that lower rates of loneliness of individuals in relationships might be caused by the availability of physical contact^13^. Similarly, singles were found to receive stronger benefits to their mood than individuals in a relationship when embracing^15^. Further supporting Heatley Tejada et al.’s conclusion, the amount of daily touch within one’s relationship has been found to predict well-being six months later, with the amount of touch positively associated with well-being^22^. Meanwhile, another study found that lower frequencies of touch in relationships, at least partially, account for an association with poorer well-being^22^. The effects of touch on well-being also applied to individuals avoidant of touch, suggesting touch in a relationship benefits both those who avoid and crave touch^23^. Debrot et al. also suggested that physical closeness within a relationship leads to psychological closeness^22^. Although not specifically touch, higher romantic involvement has been related to lower feelings of loneliness^24^. When inferring that physical and psychological closeness equate to romantic involvement, it could suggest that increased touch in a relationship may relate to lower feelings of loneliness. To date, however, the idea of lower feelings of loneliness in non-single individuals relating to increased access to touch has not been explicitly investigated.

The scope of touch in and out of relationships is enormous. Affectionate touch is prevalent worldwide, with most of it occurring with partners and their children^25^. Relatedly, affectionate relationships with their children have been found to buffer loneliness in older parents^26^. However, the association between relationships and loneliness loses its significance when high friendship closeness is included^24^. The relationship of friendship closeness with loneliness is like that of romantic involvement, with higher closeness relating to lower feelings of loneliness^24^. In sum, multiple factors interact with loneliness, including, but not limited to, touch with a spouse and children and the broader relationships with family and friends.

In addition to touch and relationship status, personality has also been strongly connected to loneliness. Both emotional stability(opposite of neuroticism) and extraversion are significantly associated with loneliness^27^. Additionally, emotional stability may moderate extraversion; people high in extraversion and low in emotional stability show lower levels of life satisfaction^28^. Moreover, emotional stability has been positively associated with daily mood^15^. This suggests that individuals high in extraversion and low in emotional stability may be more prone to loneliness. These individuals may also react more to touch, as extraverts enjoy social interaction, while low emotional stability limits contact^28^.

The perspectives reviewed above suggest that feelings of loneliness may correlate with interpersonal contact, but the relationship with physical touch is particularly important, especially among single individuals. An experiment and survey were created to investigate further the claims that increased availability of touch to individuals in relationships relates to lower feelings of loneliness and to test if the benefits of touch are transferable to video calls.

The experiment measured self-reported loneliness before and after a conversation via video call. We compared changes in loneliness scores among three experimental groups. In one condition, the call took place without the camera (audio-only). In the other conditions, both audio and cameras were activated; these conditions differed only in that, at the end, the call ended in a “virtual high-five” to simulate touch. In parallel to the primary investigation, we included other questions relevant to loneliness. We predicted that individuals with more physical touch in their relationship would score lower on the loneliness scale and that individuals subject to the simulated touch would significantly decrease feelings of loneliness than those who were not.

## Methods

### Participants

A total of 64 participants were recruited, primarily using the participant platform Prolific (www.prolific.co). This was done by the interviewer being notified of the individual’s relationship status before the call started. Prolific participants received a small incentive payment (approximately £2.35).

Participants were randomly assigned to one of three experimental groups (call without video, call with video, call with video, and simulated touch), with measures in place to balance the number of participants in each relationship group and condition. Three participants were removed from the study due to failing attention checks and a technical error. A fourth participant was removed (before analysis) because she reported being a widow, which would likely make her an outlier. The distribution of gender and relationship status across the conditions can be seen in Table 1. The final sample comprised 60 participants (31 males, 28 females, 1 nonbinary), averaging 31 years of age (median = 27, SD = 12.6). A total of 32 (53%) participants were in a relationship, and 28 (47%) were single. One participant completed the survey but did not take part in the call. The country of residence was not officially recorded but was discussed. World regions included Africa, Europe, and the Americas.

**Table 1:** Distribution of participants’ gender and relationship status by experimental condition (*F* Female, *M* Male, *NB* non-Binary).

Condition	Relationship status		Total
	Single	In a relationship	
No video	9 (5 F, 4 M)	10 (5 F, 4 M, 1 NB)	19
Video without touch	10 (6 F, 4 M)	10 (3 F, 7 M)	20
Video with simulated touch	8 (3 F, 5 M)	12 (6 F, 6 M)	20

One participant in the No video condition withdrew from the experimental part of the study.

### Procedure and materials

Participants were recruited under the pretext that the study was about “different factors in everyday life affecting individual’s well-being and the mechanisms to help cope with them.” They then used an online scheduler to reserve an appointment for the call. The experimenter then emailed links to both the online survey and video call (using Zoom) just before their scheduled appointment.

The survey was created using Qualtrics and was split into two parts, with a question requiring one of three passcodes, indicating which condition was applied, in between the two parts. Firstly, the survey presented participants with the information sheet and confirmation of informed consent. Next, demographic information, including age, gender, sexual orientation, working situation, and relationship status, was collected. Participants were asked about their home and social life, including quality time spent with family and time spent with friends in person and online (e.g., video call, phone call, gaming with voice chat) over the past 30 days. Quality time with family was used to differentiate between voluntarily spending time with family and cohabiting.

It should be noted that this study was conducted during the COVID-19 pandemic, while special measures remained in place, but just as many restrictions were being lifted. A slightly modified version of the UCLA Loneliness Scale (Version 3) (UCLA-LS3)^27^ was then applied, with question order randomised. Four options were available, including ‘Never’, ‘Rarely’, ‘Sometimes’, and ‘Often’; with ‘Often’ replacing Russell’s^27^ ‘Always’ for smoother reading and answering. This was also done in the UCLA-S^29^. Despite the change, this study had comparable internal reliability (Table 2). The longer UCLA-LS3^27^ was chosen over the UCLA-S^29^ for increased sensitivity. Additionally, participants were directly asked how often they feel lonely, in line with Office for National Statistics’ guidance^30^.

**Table 2:** Internal reliability scores of original publications and this study.

Scale	Author’s Cronbach alpha	This study’s Cronbach alpha
UCLA-LS3	0.89–0.94	0.91–0.95
TIPI		
Extraversion	0.68	0.80
Agreeableness	0.40	0.38
Conscientiousness	0.50	0.59
Emotional stability	0.73	0.55
Openness to experience	0.45	0.58

Following the loneliness scale, participants who indicated that they were in a relationship received questions about their relationship. These questions included distance of relationship (consisting of three answers: living together, living nearby, long-distance) and frequency of contact in their relationship. The four frequency questions asked participants over the past 30 days, how often (never, once or twice a month, multiple times a month, once a week, multiple days a week, and every day) they: had physical contact with their partner (e.g. hugging, holding hands, cuddling, etc.), had sexual contact with their partner, saw their partner in person, and had a call or video call with their partner.

The following section included the Ten Item Personality Index (TIPI)^31^, which assessed the Big Five personality types. Internal reliability was comparable in all personality factors except for Emotional Stability, which had a lower Cronbach’s Alpha (Table 2). The TIPI has been found to fit the Five-Factor Model well and is recommended for use when minimising fatigue^32^. The next page of the survey asked participants to join the Zoom call, where the code to continue would be given.

The experiment was conducted in the call, which took the form of an informal conversation leading call times to vary widely. The conversation consisted of small talk and was largely participant-led; however, the researcher had an interview guide if needed (Supplementary Methods S1). The researcher wore the same attire for every call to control for variables across calls. Participants were subject to a call with one of three conditions: a call with no video and initials showing, a call with video, and a call with a high five towards the camera at the end. The third condition was counted as the virtual/simulated touch. A ‘high-five’ was chosen for the ease of use over video and social acceptance. A hug was considered, as it is more affectionate, but it was judged to be impractical with a computer and less likely to be performed by strangers. Near the end of the call, participants were sent a code through Zoom, identifying which condition was applied. When explaining part 2, participants were told there were a few more questions about how they felt. When entered, this code allowed participants to move on to part 2 of the survey. Once participants started part two, the call ended.

Part 2 of the survey consisted of another copy of the UCLA-LS3. The order of questions was randomised again. The survey concluded with a debriefing page, informing participants about the purpose of the study and giving resources for mental health support.

### Ethical considerations

All research was conducted following British Psychological Society ethical guidelines and approved by the General University Ethics Panel of the University of Stirling. Several steps were taken to ensure anonymity. During the study, Zoom calls were not recorded and were kept confidential. Any records of emails were deleted and purged from Calendly and Outlook after the study. Participants were given a random ID, which they could use to withdraw their data up to 10 days after participating. As there were potentially sensitive topics in the survey, participants were asked not to participate if uncomfortable discussing their mental health, social or romantic life. Participants could withdraw at any time with no reasons given. A debrief sheet was also provided with mental health resources in multiple countries for anyone who unexpectedly felt distressed.

### Data analysis

Power analysis indicated that 60 participants were required, based on a predicted Cohen’s d effect size of 0.8 and power of 0.8 calculated for the omnibus ANOVA. Possible relationships between different factors (each Big Five personality trait, quality time with family, time with friends in person, and time with friends online) and loneliness were investigated through a series of correlations with loneliness scores. This determined which factors were retained for further analysis. A Shapiro–Wilk normality test showed that only the loneliness and emotional stability scores were normally distributed, leading to all correlations using Spearman’s Rho^33^. Additionally, a t-test and ANOVA were run between loneliness scores and relationship status and loneliness scores and living situation, respectively.

To investigate the relationship between the frequency of touch in a relationship and feelings of loneliness, only participants in a relationship (n = 32) were analysed. An additional participant was removed due to missing information on sexual contact. We calculated a measure of relationship touch by adding the scores of frequencies of sexual and non-sexual touch and dividing them by two (Cronbach alpha = 0.76). Because these measurements were not normally distributed, Spearman’s Rho correlations were conducted on each relationship contact item (non-sexual touch, sexual touch, seeing their partner in person, calling their partner) and the relationship touch scale.

A one-way ANOVA was fit to the data to determine the relationship between the distance of a relationship and loneliness. We also used one-way ANOVA to compare loneliness among single, low-touch non-single, and high-touch non-single participants. High and low touch groups were determined based on the median of the touch scale.

Finally, the effect of a call and simulated touch on loneliness was investigated through a paired samples t-test and a linear mixed-effects model. To run these analyses, the before and after scores were grouped. The paired samples t-test determined any difference in loneliness score before and after the call, regardless of which condition. To determine a possible influence of simulated touch on loneliness, a mixed-effects model was used to compare loneliness scores before and after each call; additional covariates included relationship status, and participant ID was included as a random effect.

## Results

### Preliminary analyses

A significant, negative correlation was found between extraversion and loneliness scores r_s_(58) = − 0.61, p < 0.001, and emotional stability and loneliness scores r_s_(58) = − 0.42, p = 0.001. While agreeableness, openness, and conscientiousness were not significantly correlated with loneliness scores (Table 3). These correlations helped direct future analyses.

**Table 3:** Additional correlations with loneliness.

Item correlating to loneliness	Spearman’s rho (r_s)_	Significance (two-tailed) (p-value)	N
Extraversion	− 0.61	**< 0.001**	60
Agreeableness	− 0.22	0.099	60
Openness	− 0.18	0.171	60
Conscientiousness	− 0.21	0.113	60
Emotional stability	− 0.42	**0.001**	60
Quality time with family	− 0.28	**0.031**	60
Time with friends in person	− 0.27	**0.041**	60
Time with friends online	− 0.02	0.900	60

Significant values are in bold.

Further correlation results between loneliness scores and quality time with family, time with friends in person, and online are in Table 3. Quality time with family and time with friends in person were significantly and negatively correlated with feelings of loneliness. However, both relationships were of small effect. In contrast, time with friends online was not significantly correlated with loneliness score. A one-way ANOVA revealed no significant relationship between living situation and feelings of loneliness (*F*(9,57) = 1.44, p = 0.196; *d* = 1.03).

### Frequency of touch in relationships

An initial independent t-test found that the 32 participants in a relationship (M = 43.38, SD = 10.38) had a lower loneliness score than the 28 single participants (M = 50.11, SD = 10.34), *t*(58) = − 2.51, p = 0.015; *d* = 0.64.

Loneliness scores and frequency of touch in a relationship were significantly and negatively correlated r_s_(29) = − 0.43, p = 0.017. A post hoc analysis, looking at each correlation of physical contact (r_s_(29) = − 0.36, p = 0.048) and sexual contact (r_s_(29) = − 0.35, p = 0.052) within the touch scale, found a marginally significant, medium, negative correlation between physical contact and loneliness, and a marginally non-significant, medium, negative correlation between sexual contact and feelings of loneliness. Results for all relationship frequency questions can be found in Table 4. A one-way ANOVA revealed no significant relationship between relationship distance and feelings of loneliness (*F*(2,29) = 1.40, p = 0.263; *d* = 0.63).

**Table 4:** Frequencies in a relationship correlating with loneliness.

Item correlating to loneliness	Spearman’s Rho (r_s)_	Significance (two-tailed) (p-value)	N
Physical contact	− 0.38	**0.032**	32
Sexual contact	− 0.35	0.052	31
See partner in person	− 0.28	0.123	32
Call partner	− 0.05	0.785	32

Significant values are in bold.

Frequency was measured over the previous 30 days.

**Figure 1:**
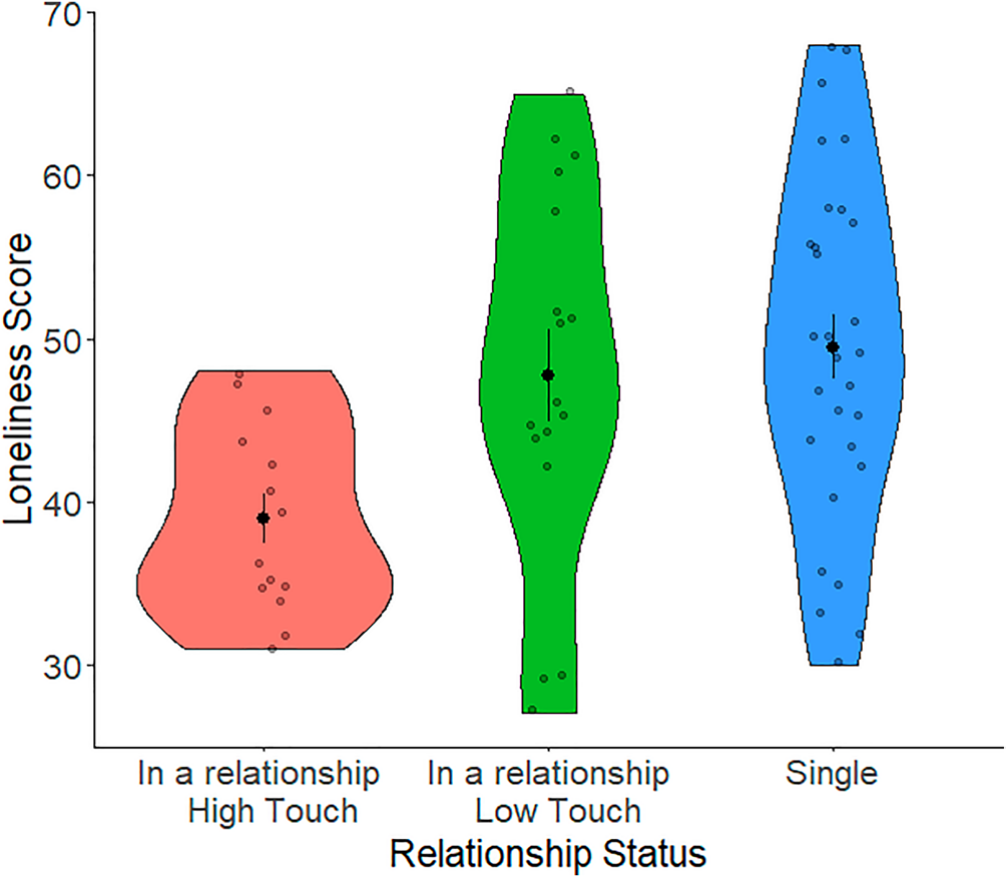
Mean and dispersion of loneliness scores by relationship status (in a relationship with high touch, in a relationship with low touch, or single).

Another one-way ANOVA revealed significant differences in loneliness scores (*F*(2,57) = 5.32, p = 0.008; *d* = 0.88) among single participants, those in a low-touch relationship, and those in a high-touch relationship. Normality tests on the residuals met assumptions for normality. After accounting for the familywise error rate, post hoc t-tests indicated no significant difference in loneliness score means between single participants (mean = 49.48, SD = 10.70) and participants in low-touch relationships (mean = 47.71, SD = 11.68). However, a significant difference in loneliness score means was found between participants in high-touch relationships (mean = 38.92, SD = 5.73) and both single participants and those in low-touch relationships (Fig. 1).

**Figure 2:**
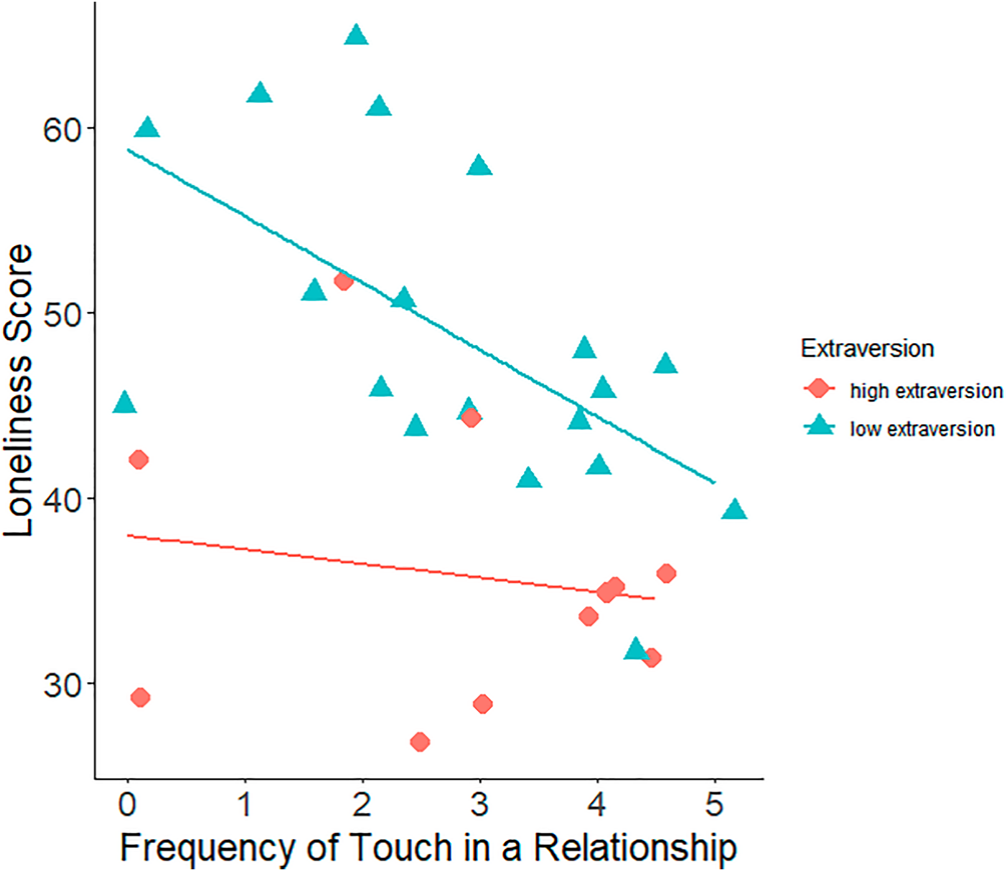
Relationship between frequency of touch and loneliness score, split by extraversion.

A linear regression testing the moderation of the relationship between touch and loneliness through extraversion found that the model explained 53.58% of the variance (R^2^ = 54, *F*(3,27) = 10.39, p < 0.001, *f*^2^ = 0.40). There was a significant, negative main effect of frequency of touch in a relationship on loneliness scores, β = − 0.32, t = − 2.40, p = 0.023. Extraversion also had a significant, negative main effect on loneliness scores, β = − 0.55, t = − 4.15, p < 0.001. The interaction between touch in a relationship and extraversion was also significant, β = 0.28, t = 2.10, p = 0.045, such that the negative association between frequency of touch in a relationship and loneliness scores were greater at low levels of extraversion (Fig. 2).

### Effect of simulated touch via video call on loneliness

A paired-samples t-test found a significant difference between loneliness scores before (mean = 46.56, SD = 10.90) and after (mean = 44.63, SD = 12.64) the call, *t*(58) = 3.38, p = 0.001, *d* = 0.44.

To investigate this further, we used a mixed-designs ANOVA with loneliness scores before and after the call as the within-subjects factor, and with the condition (no video, video, video with simulated touch) and the relationship type (high-touch, low-touch, single) as between-subject factors. As expected, the main within-subjects effect of the call was significant, but there were no significant interactions or main effects of conditions (Table 5). The only other significant effect was the main effect of the relationship type (p = 0.020).

**Table 5:** Results of mixed-effects ANOVA.

Term	*F*	df	*p*
Change in loneliness before/after the call	11.68	1.50	**0.001**
Change in loneliness × condition	0.16	2.50	0.855
Change in loneliness × relationship type	0.28	2.50	0.754
Change in loneliness × condition × relationship Type	0.55	4.50	0.699
Condition	0.16	2.50	0.849
Relationship type	4.25	2.50	**0.020**
Condition × relationship type	0.81	4.50	0.527

Significant values are in bold.

Changes in loneliness before and after the call (above) and between-subjects differences in loneliness (below).

When we reran the model, this time including as covariates the effects found previously to significantly correlate with loneliness (i.e., extraversion, emotional stability, quality time with family, and time with friends in person), the only significant terms were, again, relationship type (F2,46 = 4.75, p = 0.013), emotional stability (F1,46 = 5.15, p = 0.028) and extraversion (F1,46 = 14.29, p < 0.001). Again, this analysis had no effect of Condition, either as a main effect or interaction.

## Discussion

To the researchers’ knowledge, this is the first study looking directly at the effects of simulated touch over video calls on feelings of loneliness. This study additionally contributed to the existing literature on the relationship between touch in a relationship and feelings of loneliness. Reaffirming Heatley Tejada et al.’s^10^ conclusion, the increased frequency of touch in a relationship negatively correlated with feelings of loneliness. Additionally, extraversion was found to play a significant role in how much touch lowered loneliness in a relationship. Contrary to prediction, there were no significant differences in the reduction of loneliness scores among having no video, video, and video with ‘simulated touch’; however, the call itself had a small but significant effect on decreasing feelings of loneliness.

Concurrent with previous research, extraversion and emotional stability (opposite of neuroticism) were found to have significant relationships with feelings of loneliness. Overall, individuals higher in either extraversion or emotional stability scored lower in feelings of loneliness than people lower in extraversion or emotional stability. Finally, the strong relationship between extraversion and loneliness and the high Cronbach’s alpha indicated that extraversion could be used in later analyses.

This study supports Heatley Tejada et al.’s claim that the decreased feelings of neglect in non-single individuals were due to the availability of touch in the relationship^13^. Firstly, participants in a relationship scored significantly higher on the UCLA-LS3 than single participants. This confirmed predictions and suggestions by Heatley Tejada et al.^13^. Secondly, touch’s role in loneliness is so significant that the loneliness scores of people in low-touch relationships were closer to those of single participants than those in high-touch relationships. When combined with Heatley Tejada’s results, this study suggests that touch plays more of a role in feelings of loneliness than the relationship status itself. It should also be noted that the type of touch participants was asked about was affectionate (e.g. hugging, holding hands, cuddling) and sexual contact. Although they do not explicitly mention affectionate touch to participants, the examples of contact given match the description of Jakubiak and Feeney’s definition of affectionate touch^10^. The frequency of affectionate touch was only marginally significant, while sexual touch fell just shy of the threshold for significance. Both types of touch had a similar relationship with loneliness, but the combination had a much stronger negative relationship with loneliness. This suggests that a combination of sexual and affectionate touch is vital in lowering feelings of loneliness within relationships. Since the combination of sexual and affectionate touch combined had the strongest negative correlation, it is important to look at more than one factor in touch when measuring its relationship with loneliness.

Additionally, quality time with family and time with friends in person were both negatively associated with loneliness, emphasising the importance of interpersonal relationships in regulating loneliness. This was not true for seeing one’s partner in person. The distance of a relationship also had no significant relationship with loneliness. This further supports the claim that relationships regulate loneliness through physical touch rather than relationship status directly influencing loneliness.

In addition to the relationship between the frequency of touch and loneliness, extraversion was found to moderate the relationship. This moderation was partly supported by previous literature. The moderation was opposite to predictions based on Fadda and Scalas’s^28^ suggestion that extraverts prefer social contact. This literature suggested that people high in extraversion would be more reactive to touch, as it is craved more. In this study, participants low in extraversion appeared more reactive to touch. This is illustrated in Fig. 2, where the difference in feelings of loneliness between high and low frequency of touch was noticeably greater in introverts than in extraverts. This supports Debrot et al.^23^, and highlights the importance of touch in a relationship irrespective of extraversion. It also emphasises the importance of including extraversion in any research about touch and loneliness. In speculation, the reason behind the negative relationship between extraversion and loneliness observed by Russell^27^ might not be the extraversion itself but rather the assumption that introverts seek less physical contact and therefore have less physical contact.

The experiment tested if simulated touch in a video call reduces feelings of loneliness. An initial analysis found a slight decrease in feelings of loneliness after the call, regardless of the condition. However, there was no significant change among the conditions (no video, video, and/or video with simulated touch). Therefore, it appears that the effects of touch on loneliness are not transferable to a simulated touch during a video call, at least in the design we used.

## Limitations and future directions

Although we tried to standardise the nature of the call as far as possible, there remained differences across calls, perhaps notably the time spent on the call. This was because some participants were simply much chattier and happy to talk for long periods, while others only gave extremely brief responses. Unfortunately, the length of the call was not recorded, but this would be done in any future study. Participants could also have been asked how connected with the researcher they felt after the call. The emotional stability analysis was also flawed due to a divergent and low Cronbach’s alpha compared to Gosling et al.’s benchmark^31^. Using “quality” time with family but not friends could have confused participants due to inconsistent language. Furthermore, the assumption that quality time needs distinction in family relationships but not friendships was a mistake. Studies looking at time with friends and family should also look at multiple aspects of both.

Future research should investigate the differences between affectionate and non-affectionate touch. In our study, we chose to use a ‘high five’ as the simulated touch, and different results may have been found if an action such as hugging the camera was used. Indeed, other studies of loneliness and touch, such as those by Heatley Tejada et al.^13^ and Jakubiak and Feeney^10^, investigated affectionate touch. This difference could be an important factor in the reduction of loneliness. In our study, the questions regarding touch in a relationship were also focused on affectionate touch, and this could explain why we found significant relationships between touch and loneliness in the questionnaire responses but not in the experiment.

Finally, the long-term effects of having calls on loneliness should be investigated. While there was no significant effect of simulated touch during the call, there was an overall reduction in loneliness score following the call, supporting the findings of Petersen et al.^7^. Although we do not know how long these effects may last, we may deduce that they are quite limited in length because, as shown in our questionnaire responses, there was no significant relationship between loneliness and the frequency of digital interactions (online/call) with friends or romantic partners. Therefore, calls appear to give immediate, but short-term, relief to feelings of loneliness.

## Conclusions

In conclusion, our data show that an increase in the frequency of touch within a relationship relates to markedly lower feelings of loneliness, along with personality characteristics such as extraversion and emotional stability. On the other hand, while a call can itself significantly reduce loneliness scores, simulated touch during a video call has no significant benefit (at least in our design). Importantly, the longevity of this reduction is unknown, as there was no significant association between the frequency of calls and feelings of loneliness. Future research may find that different contexts and types of simulated touch prove to be more effective, but for now, our study shows that, if feeling lonely, a call of any type can quickly reduce these feelings.

## Supplementary Information

Supplementary Information.

## Data Availability

The datasets used and analysed during the current study are available from the corresponding author on reasonable request. Alternatively, the datasets can be accessed at DataSTORRE: Stirling Online Repository for Research Data, http://hdl.handle.net/11667/207. Data in the repository may require a request to be submitted if accessing it before the end of the embargo period. Code availability for R-Studio is available upon request.
