# Supplementary material for: An investigation of simulated and real touch on feelings of loneliness
Source: Sci Rep. 2023 Jun 30;13:10587. doi: 10.1038/s41598-023-37467-5 (PMC10313660; doi:10.1038/s41598-023-37467-5)
Supplement: Supplementary file 1 — Supplementary Information. [file 41598_2023_37467_MOESM1_ESM.pdf]

An investigation of simulated and real touch on feelings of loneliness

Nicholas L.T. Gray, S. Craig Roberts

Supplement Methods 1

Call Guide

1. Introduce self as [redacted] and ask their name
2. Ask where in the world they are
    a. Expand on this: ask about weather, what it is like, etc.
3. Ask how they have gotten on with the study
4. Explain there is just one more task, not long left
5. Administer condition (high five) if appropriate
6. Send code correlating with given condition
7. Once participants confirm the next questionnaire has appeared
    a. thank them and end call
